# Interferon Regulatory Factors as Potential Therapeutic Targets in Cardiovascular Disease: Focusing on Vascular Inflammation

**DOI:** 10.3390/ijms27177647

**Published:** 2026-08-26

**Authors:** Chenxi Bai, Weixu Liu, Yubo Wang, Huixia Zhu, Xing Fan

**Affiliations:** Department of Biochemistry and Molecular Biology, School of Medicine, Nantong University, Nantong 226001, China

**Keywords:** interferon regulatory factors, vascular inflammation, cardiovascular disease, atherosclerosis, precision intervention, targeted therapy

## Abstract

The interferon regulatory factor (IRF) family exerts dual regulatory roles in vascular inflammation. Pro-inflammatory IRF1/3/5/7 drive endothelial dysfunction, macrophage M1 polarization, vascular smooth muscle cell (VSMC) transdifferentiation, and adaptive immune amplification via nuclear factor-κB (NF-κB), NOD-like receptor pyrin domain-containing 3 (NLRP3), cyclic GMP-AMP synthase-stimulator of interferon genes (cGAS-STING), and Janus kinase/signal transducer and activator of transcription (JAK/STAT) pathways. Conversely, IRF4/8 mediate anti-inflammatory effects by promoting M2 polarization, reverse cholesterol transport, and dendritic cell regulation. In atherosclerosis, IRFs display spatiotemporal specificity: endothelial IRF3 in early stages, macrophage IRF1/5 in mid-stages, and smooth muscle IRF7/IRF8 in late stages, supporting phase-specific precision interventions—early IRF3 blockade, mid-stage modulation of IRF5/IRF4 balance, and late combined inhibition of IRF7/8 to stabilize plaques. IRFs also critically participate in hypertensive remodeling, acute coronary syndrome, heart failure, and aortic aneurysm through conserved innate and adaptive immune axes, highlighting their potential as cross-disease biomarkers and therapeutic targets. Major challenges include network redundancy and functional compensation, necessitating single-cell multi-omics and targeted delivery systems for spatiotemporally precise, individualized modulation.

## 1. Introduction

### 1.1. Global Epidemiology and Disease Burden of Cardiovascular Disease and Atherosclerosis (AS)

Cardiovascular disease (CVD) has consistently ranked as the leading cause of global disease burden and remains the primary driver of mortality and disability worldwide [1]. The World Health Organization’s Noncommunicable Diseases Country Profiles 2018 reported that CVD accounts for over 17 million deaths annually, representing 31% of total global mortality, surpassing other chronic diseases such as cancer and respiratory illnesses [2]. AS, as the common pathological substrate underlying fatal cardiovascular and cerebrovascular events including coronary heart disease, ischemic stroke, and peripheral artery disease, operates as a continuous process throughout disease progression [3]. Accelerated global population aging, the widespread adoption of high-fat and high-sugar diets, increasingly sedentary lifestyles, and the rising prevalence of metabolic risk factors such as hypertension, diabetes, and obesity have driven a sustained increase in AS incidence, with a notable trend toward earlier onset [4]. The growth rate of AS-related diseases is particularly pronounced in low- and middle-income countries, posing a significant public health challenge [5]. The progression of AS from early fatty streaks to unstable, vulnerable plaques, culminating in rupture, erosion, and thrombosis, precipitates acute myocardial infarction, stroke, and even sudden cardiac death [6]. Its high incidence, recurrence rate, disability rate, and mortality impose substantial economic and healthcare burdens on families and society. Consequently, there is an urgent clinical need and significant scientific value in elucidating the pathogenesis of AS, identifying novel therapeutic targets, and formulating precise prevention and treatment strategies [5].

### 1.2. Etiology of Atherosclerosis and the Plurality of Pathogenic Hypotheses

The pathological mechanisms underlying AS are highly complex, involving multi-cellular interactions, activation of multiple signaling pathways, and regulation by numerous factors [7]. Classical theories have been proposed to systematically explain its initiation and progression. The lipid infiltration hypothesis, the earliest proposed, posits that abnormal elevation of plasma low-density lipoprotein cholesterol (LDL-C) and its infiltration into the arterial intima constitute the core initiating event of AS, laying the theoretical foundation for lipid-lowering therapy [8]. The endothelial injury response hypothesis further suggests that stimuli such as abnormal hemodynamic shear stress, oxidized lipids, toxins, and inflammatory cytokines lead to endothelial barrier dysfunction, inducing adhesion molecule expression, monocyte recruitment, and initiating a cascade of inflammation-repair imbalance [9]. The inflammation hypothesis explicitly characterizes AS as a chronic inflammatory disease, emphasizing that disorders of innate and adaptive immunity drive plaque formation, progression, and rupture throughout the disease course [10]. The oxidative stress hypothesis focuses on excessive reactive oxygen species (ROS) generation, which causes lipid oxidation, protein modification, DNA damage, and apoptosis, amplifying inflammation and lipotoxicity [11]. Furthermore, hypotheses including abnormal shear stress, gut microbiota dysbiosis, epigenetic dysregulation, and aberrant cell death continue to enrich the theoretical framework of AS [12,13,14]. The interplay and synergy of these diverse mechanisms constitute a multifaceted etiological network, providing multiple levels of targets for therapeutic intervention [15].

### 1.3. Central Role of Vascular Inflammation in Atherosclerosis and Its “Double-Edged Sword” Nature

Vascular inflammation serves as the central hub linking risk factors to the pathological progression of AS, spanning the entire continuum from endothelial injury, lipid deposition, foam cell formation, plaque growth, and destabilization to plaque rupture and thrombosis [16]. Recent studies have confirmed that vascular inflammation exhibits a typical “double-edged sword” property: under physiological conditions, an appropriate and controlled inflammatory response contributes to vascular homeostasis, clears injurious agents, promotes endothelial repair, and regulates cellular metabolism and survival, thereby exerting vasoprotective effects [17]; conversely, under pathological conditions, chronic, persistent, and uncontrolled low-grade inflammation continuously activates signaling axes such as NF-κB, NLRP3, and JAK/STAT, promoting macrophage infiltration and foam cell formation, abnormal proliferation and migration of smooth muscle cells, degradation of the extracellular matrix, and significantly reducing plaque stability, ultimately triggering acute cardiovascular and cerebrovascular events [18,19]. This duality suggests that anti-inflammatory intervention in AS should not be a simplistic “blanket anti-inflammation” approach, but rather should achieve spatiotemporally precise, intensity-appropriate, and target-specific regulation to restore vascular inflammatory homeostasis [20].

### 1.4. Overview of the Interferon Regulatory Factor (IRF) Family

#### 1.4.1. Family Members and Structural Features

Interferon Regulatory Factors (IRFs) constitute a highly conserved family of transcription regulators, with nine members (IRF1 to IRF9) identified in mammals [21]. IRF family proteins share a highly conserved domain architecture: the N-terminal DNA-binding domain (DBD) contains a characteristic helix-turn-helix (HTH) motif that specifically recognizes and binds to the interferon-stimulated response element (ISRE) sequence [21]; the C-terminal regulatory domain mediates protein dimerization, nuclear translocation, and interactions with co-activators or co-repressors, thereby determining transcriptional activation or repression functions [22]. This unique structure enables IRFs not only to regulate interferon (IFN) transcription but also to participate extensively in immune cell differentiation, inflammatory signal transduction, cell cycle regulation, apoptosis, and metabolic reprogramming [23].

#### 1.4.2. Physiological Functions and Pathological Roles

The IRF family serves as a core regulatory molecule of both innate and adaptive immunity, playing critical roles in antiviral infection, immune tolerance, tumorigenesis, and inflammatory diseases [24]. Recent studies have confirmed that IRFs are widely expressed in key AS-pertinent effector cells, including vascular endothelial cells, smooth muscle cells, macrophages, and dendritic cells, and are involved in vascular homeostasis maintenance, amplification of inflammatory signals, reprogramming of lipid metabolism, and cell fate determination [25]. In the context of vascular inflammation and AS, distinct IRF members exhibit opposing regulatory effects: IRF1, IRF3, IRF5, and IRF7 primarily exert pro-inflammatory effects, whereas IRF4 and IRF8 possess anti-inflammatory, vasculoprotective, and plaque-stabilizing properties [26,27,28]. This bidirectional regulatory feature positions IRFs as ideal candidate targets for precision intervention in vascular inflammation [29].

### 1.5. Purpose and Framework of This Review

Several recent reviews have provided comprehensive overviews of the roles of IRFs in atherosclerosis and ischemia/reperfusion injury, establishing an important foundation for understanding their cell-specific and context-dependent functions [25,30,31]. Building on these studies, the present review focuses on several complementary aspects. First, we consider IRF signaling from a spatiotemporal perspective, highlighting how the contribution of different IRF members changes across the progression of vascular inflammation, from endothelial activation to macrophage remodeling and advanced plaque instability. Second, we extend the discussion beyond atherosclerosis to other major cardiovascular diseases, including hypertension, acute coronary syndrome, heart failure, and aortic aneurysm/dissection, with the aim of identifying both conserved and disease-specific IRF regulatory patterns. Third, we place these mechanistic insights in a translational context by discussing potential strategies for targeted delivery and combination intervention, together with the challenges posed by network redundancy and the risk of systemic immunosuppression. Collectively, these perspectives provide an integrated view of IRF signaling in cardiovascular inflammation and highlight potential directions for more precise therapeutic intervention.

## 2. Vascular Inflammation and Atherosclerosis

### 2.1. Fundamental Concepts and Duality of Vascular Inflammation

Vascular inflammation is a defensive response of the arterial wall to injury, lipids, infection, and metabolic disturbances, and it serves as a core driver of AS [16]. Depending on the intensity, duration, and resulting effects, vascular inflammation exhibits dual protective and injurious functions: acute, moderate, and self-limiting inflammation can clear harmful substances, promote tissue repair, inhibit plaque formation, and enhance plaque stability [32]; conversely, chronic, persistent, low-grade inflammation, through the sustained release of inflammatory cytokines, chemokines, and matrix metalloproteinases, disrupts vascular architecture, expands the necrotic core, weakens the fibrous cap, and accelerates plaque instability and rupture [33]. Dysregulation of vascular inflammatory homeostasis is a hallmark of AS initiation, and its duality dictates that anti-inflammatory strategies must evolve toward precision and individualization [34].

### 2.2. Pathological Features of Chronic Vascular Inflammation in AS

#### 2.2.1. Synergistic Roles of Vascular Endothelial Cells, Smooth Muscle Cells, and Macrophages

**Figure 1:**
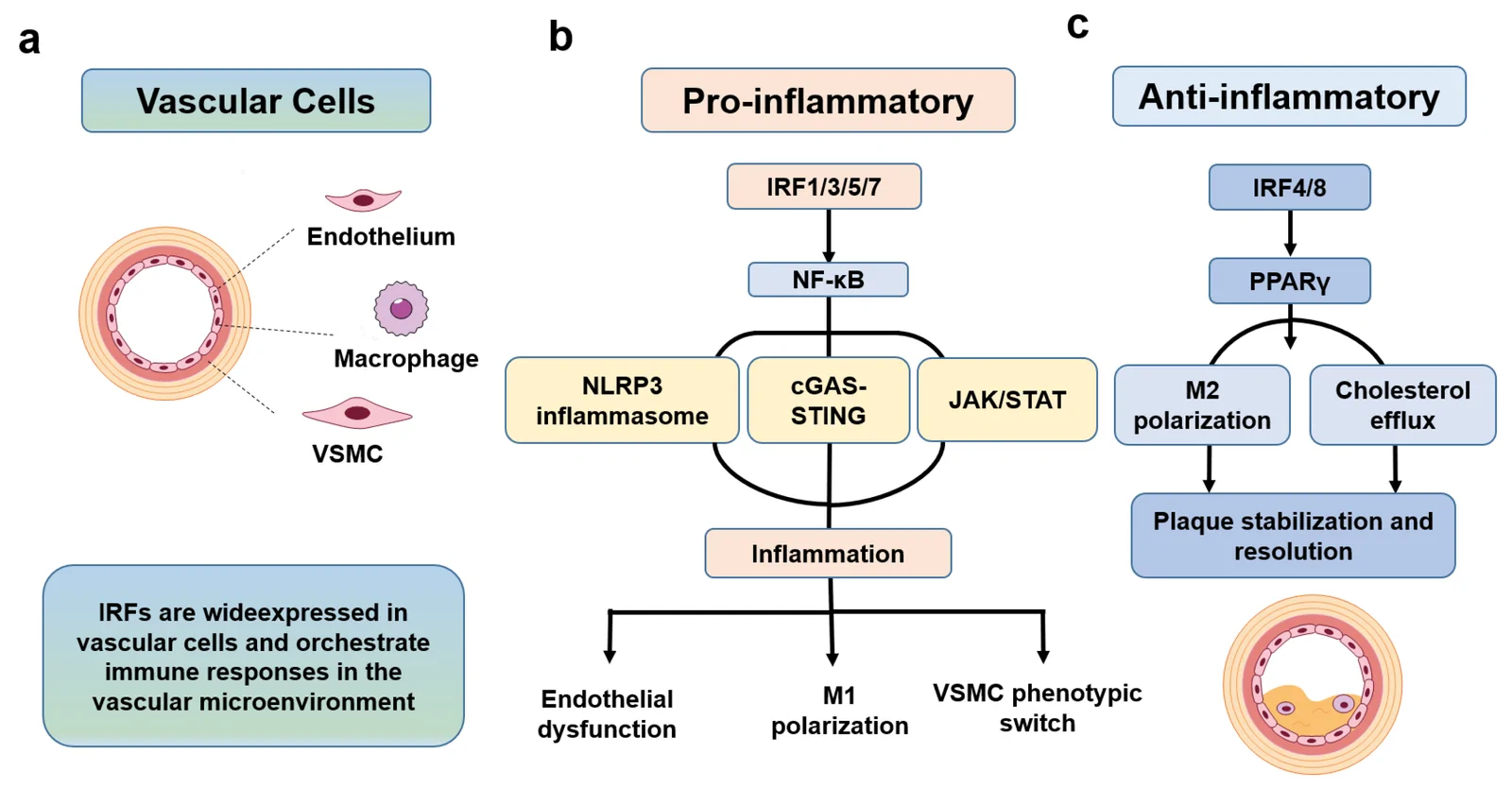
Interferon Regulatory Factors Differentially Regulate Atherosclerosis via Two Opposing Inflammatory Arms. (**a**) IRFs are expressed in endothelial cells, macrophages and VSMCs to regulate vascular immune responses. (**b**) IRF1/3/5/7 activates NF-κB and downstream inflammatory pathways, inducing endothelial dysfunction, M1 macrophage polarization and VSMC phenotypic switch to aggravate atherosclerosis. (**c**) IRF4/8 upregulates PPARγ, promotes M2 polarization and cholesterol efflux, thus stabilizing atherosclerotic plaques and resolving inflammation. Created with Microsoft PowerPoint.

Chronic inflammation in AS is driven cooperatively by multiple vascular wall cells, forming a progressively amplified pathological cycle: ① Endothelial injury and activation: Risk factors increase endothelial permeability and upregulate VCAM-1, ICAM-1, and E-selectin, mediating monocyte rolling, adhesion, and migration [35]. ② Macrophage infiltration and foam cell formation: Monocytes differentiate into macrophages, which extensively engulf oxidized low-density lipoprotein (ox-LDL) via scavenger receptors, forming foam cells and constituting early lipid streaks [36]. ③ Abnormal activation of smooth muscle cells: Medial smooth muscle cells migrate to the intima, proliferate, secrete collagen and matrix, and simultaneously release inflammatory cytokines to amplify the inflammatory response [37] (Figure 1). These three cell types interconnect through signals such as TNF-α, IL-1β, IL-6, and MCP-1, forming an inflammatory network that collectively drives AS progression [7].

#### 2.2.2. Active Interaction Between Vascular Inflammation and Atherosclerotic Plaques

AS plaques (lipid core, fibrous cap, calcification, necrotic core) engage in a bidirectional positive-feedback loop with vascular inflammation: inflammatory signals promote foam cell apoptosis and secondary necrosis, enlarge the lipid core, and activate matrix metalloproteinases (MMPs) that degrade the fibrous cap, rendering the plaque unstable [38]; plaque rupture, release of cholesterol crystals, and spillage of necrotic debris further activate the NLRP3 inflammasome and innate immunity, amplifying local and systemic inflammation and forming a vicious cycle [39]. This mechanism constitutes the core pathological basis of acute coronary syndrome and ischemic stroke [40].

#### 2.2.3. Eicosanoid Signaling in Vascular Inflammation and Atherosclerosis

Eicosanoid signaling constitutes an important lipid-derived inflammatory pathway that complements cytokine-, pattern-recognition receptor-, and inflammasome-mediated mechanisms in atherosclerosis. Arachidonic acid released from membrane phospholipids is metabolized through cyclooxygenase (COX) and lipoxygenase (LOX) pathways to generate prostanoids and leukotrienes, respectively. Among these mediators, leukotrienes, particularly leukotriene B4 and cysteinyl leukotrienes, can promote leukocyte recruitment, endothelial activation, and amplification of local inflammatory responses. Increasing experimental and clinical evidence supports a role for leukotriene signaling in the initiation and progression of atherosclerotic cardiovascular disease [41].

Thromboxane A2, another bioactive product of arachidonic acid metabolism, contributes to the inflammatory and thrombotic milieu by promoting platelet activation and vasoconstriction. In patients with peripheral arterial occlusive disease undergoing percutaneous transluminal angioplasty, elevated preprocedural urinary 11-dehydro-thromboxane B2 levels, a major stable metabolite of thromboxane A2, were associated with an increased risk of subsequent adverse cardiovascular outcomes, including death, myocardial infarction, and stroke, during follow-up [42]. These findings highlight eicosanoid signaling as a potential link between lipid metabolism, inflammatory cell recruitment, platelet activation, and thrombotic cardiovascular events.

Despite these established roles, the relationship between eicosanoid signaling and IRF-dependent inflammatory regulation remains insufficiently characterized. In particular, whether IRF-mediated transcriptional programs directly or indirectly influence eicosanoid biosynthesis, metabolism, or receptor signaling within human atherosclerotic lesions remains to be determined. Elucidating this potential crosstalk may provide further insight into the integration of lipid-derived inflammatory mediators with IRF-centered vascular inflammatory networks.

### 2.3. Current Therapeutic Strategies for AS Vascular Inflammation

#### 2.3.1. Modulation of Vascular Inflammatory Pathways

Targeting core inflammatory signaling is the central direction of current anti-inflammatory therapy for AS: the NF-κB pathway regulates the transcription of inflammatory genes; the NLRP3 inflammasome senses cholesterol crystals and damage signals, activating IL-1β maturation and release; TLR4 recognizes pathogen- and damage-associated molecular patterns; and the JAK/STAT pathway mediates the amplification of inflammatory signals [43,44,45,46]. Small-molecule inhibitors and biologics targeting these pathways have entered clinical trials, demonstrating anti-inflammatory and plaque-stabilizing potential [47].

#### 2.3.2. Regulation of Cell Survival and Death Modalities

Cellular fate directly determines plaque stability: foam cell apoptosis coupled with efficient efferocytosis can reduce the necrotic core; the balance between smooth muscle cell proliferation and apoptosis influences fibrous cap thickness; autophagy maintains cellular metabolic homeostasis; and ferroptosis exacerbates lipid peroxidation and inflammatory amplification [48,49,50,51]. Modulating cell death modalities has thus become an important strategy for plaque stabilization [52].

#### 2.3.3. Lipid-Targeted Therapies

Dyslipidemia is the fundamental risk factor for AS: statins inhibit cholesterol synthesis, PCSK9 monoclonal antibodies reduce LDL receptor degradation, PPAR modulators ameliorate the dual disturbances of lipid metabolism and inflammation, and CETP inhibitors regulate reverse cholesterol transport [53,54,55,56]. Lipid-lowering therapy not only reduces lipid burden but also attenuates vascular inflammation, improving plaque prognosis [57]. Beyond conventional LDL-C-centered therapies, lipoprotein(a) [Lp(a)] has emerged as an important contributor to residual cardiovascular risk and represents an increasingly recognized therapeutic target. Muvalaplin, an orally administered small molecule that inhibits Lp(a) formation, has demonstrated substantial and dose-dependent reductions in circulating Lp(a) concentrations in a phase 2 randomized clinical trial, with a maximal placebo-adjusted reduction of 85.8% at 12 weeks as measured by an intact Lp(a) assay [58]. However, whether such pharmacological reductions in Lp(a) translate into meaningful reductions in cardiovascular events remains to be established, and further clinical studies are needed to determine the long-term cardiovascular benefits of Lp(a) lowering with muvalaplin.

#### 2.3.4. Other Molecular Targets

Emerging targets continue to expand the therapeutic borders for AS: VEGF regulates angiogenesis; adhesion molecule blockade inhibits leukocyte recruitment; non-coding RNAs (miRNAs, lncRNAs, circRNAs) participate in post-transcriptional regulation; and epigenetic mechanisms such as DNA methylation and histone modifications influence inflammatory gene expression [59,60,61,62].

#### 2.3.5. Lifestyle Interventions

Lifestyle intervention forms the basis of primary prevention for AS: a low-fat, high-fiber diet, regular aerobic exercise, smoking cessation and alcohol restriction, weight management, and blood pressure and blood glucose control can systematically reduce inflammatory burden and risk factor exposure, delaying the onset and progression of AS [63,64,65].

## 3. Regulatory Roles of IRFs in Atherosclerosis-Related Vascular Inflammation and Initiation of Cardiac Events

### 3.1. IRFs as the “Regulatory Hub” of the Vascular Inflammatory Microenvironment

The IRF family serves as a regulatory hub of the vascular inflammatory microenvironment by integrating multiple signaling pathways, primarily the following three: (1) Integration of innate immune signaling: IRF3 and IRF7, as core transcription factors, simultaneously integrate two key innate immune signaling pathways, namely TLR and cGAS-STING. They recognize and respond to exogenous pathogen-associated molecular patterns (PAMPs, e.g., LPS, ox-LDL) and endogenous damage-associated molecular patterns (DAMPs, e.g., mtDNA, HMGB1), thereby initiating the transcription and expression of type I interferons and downstream pro-inflammatory genes, constituting the core molecular axis for the initiation and amplification of vascular inflammation [66]. (2) Integration of metabolic signaling: IRF1 and IRF5 regulate key metabolic pathways such as glycolysis and fatty acid oxidation, remodeling the energy metabolism pattern of macrophages and providing the necessary metabolic substrates and energy support for the inflammatory response, thus contributing to the sustained activation of vascular inflammation [67,68,69]. (3) Epigenetic regulation: The expression levels and transcriptional activities of IRF family members are precisely regulated by DNA methylation, histone modifications, and non-coding RNAs. These regulatory mechanisms collectively constitute the epigenetic memory of the vascular inflammatory microenvironment, enabling long-term modulation of the inflammatory response [70,71].

By virtue of their multi-signal integration capacity, IRFs can dynamically regulate the intensity and progression of vascular inflammation according to microenvironmental changes. Moreover, IRFs exhibit pronounced spatiotemporal expression characteristics across different pathological stages of AS: (1) Early stage (fatty streak): IRF3 primarily regulates vascular endothelial cell activation, inducing adhesion molecule expression via the TLR4-TRIF and cGAS-STING pathways, thereby initiating monocyte recruitment and infiltration [66,72]. (2) Intermediate stage (fibrous plaque): IRF1 and IRF5 jointly drive macrophage polarization toward the M1 pro-inflammatory phenotype and promote foam cell formation, while the anti-inflammatory effect of IRF4 is suppressed, forming a positive feedback loop that mutually reinforces inflammation and lipid deposition [28,69]. (3) Advanced stage (complex lesion): IRF7 and IRF8 participate in regulating vascular smooth muscle cell transdifferentiation and dendritic cell-mediated adaptive immune responses, exacerbating plaque calcification, fibrous cap thinning, and chronic inflammation [73,74]. This stage-specific regulatory logic, exemplified by AS, reflects the common temporal sequence of vascular inflammation from initiation, amplification, to chronicity, and its molecular framework can be extrapolated to the vascular inflammatory processes in other cardiovascular diseases.

### 3.2. IRF1

As a multifunctional transcription factor, IRF1 exerts cell type- and microenvironment-dependent dual effects in vascular smooth muscle cells (VSMCs), endothelial cells, and macrophages, serving as a central regulatory hub that drives the initiation, amplification, and persistence of vascular inflammation [30].

In VSMCs, IRF1 exhibits a dual regulatory function. On one hand, IRF1 activates the iNOS-NO pathway and upregulates the cyclin-dependent kinase inhibitor p21, inducing G1-phase cell-cycle arrest, thereby inhibiting VSMC proliferation, promoting apoptosis, limiting neointimal formation, and exerting a plaque-protective effect [75,76,77,78]. On the other hand, IRF1 directly transactivates the chemokine CCL19 and promotes the release of pro-inflammatory cytokines such as IL-1α, IL-1β, TNFα, and IL-6, enhancing VSMC proliferation and migration, thereby driving their conversion toward a pathological phenotype. The IRF1-CCL19 axis constitutes a critical pathway mediating vascular inflammation and plaque progression [78].

In vascular endothelial cells, stimulation by ox-LDL, LPS, intermittent hypoxia, and other pathological stimuli upregulates IRF1 through the NF-κB (RelB/p52) pathway, leading to the transcriptional activation of GSDMD and caspase-1, thereby directly inducing endothelial pyroptosis and disrupting the vascular endothelial barrier [79,80]. Additionally, IRF1 mediates TNFα-induced upregulation of VCAM-1, enhances leukocyte adhesion, and accelerates the maturation and release of IL-1β by promoting Caspase-1 transcription and inflammasome activation, thereby robustly amplifying endothelial inflammatory responses [81,82].

In macrophages, IRF1 is a core factor driving M1 pro-inflammatory polarization and inflammatory cell death. As a key downstream molecule of STAT1, IRF1 significantly enhances pro-inflammatory gene expression and amplifies local inflammation [83,84]. The AIM2 inflammasome can also upregulate IRF1 via the NLRC4 pathway, further reinforcing M1 polarization [85]. Under oxLDL stimulation, IRF1 promotes NLRP3 inflammasome assembly, caspase-1 activation, and GSDMD cleavage, thereby triggering macrophage pyroptosis and the subsequent release of abundant IL-1β and IL-18, which exacerbates plaque inflammation and promotes expansion of the necrotic core [86]. IRF1 also participates in the activation of the PANoptosis complex, which involves ZBP1, AIM2, and RIPK1, synergistically amplifying the inflammatory cascade [87].

IRF1-driven vascular inflammation represents the initiating step of the cardiac event chain. Regions of high IRF1 expression within plaques correspond to areas of thin fibrous caps and enlarged lipid cores, which are high-risk zones for plaque rupture [87]. Clinical evidence indicates that IRF1 expression is significantly elevated in the peripheral blood and plaque tissues of patients with acute coronary syndrome (ACS), suggesting an association between IRF1 activity and the risk of adverse cardiovascular events. Targeted inhibition of IRF1 may not only improve plaque stability but also attenuate secondary myocardial injury following ischemic events, thereby representing a potential upstream therapeutic target for interrupting the progression from plaque instability to acute cardiac events [88].

### 3.3. IRF3

IRF3 is a key regulator of endothelial inflammatory activation. Its activation primarily depends on two core pathways. In the TLR4-TRIF pathway, oxLDL and LPS recruit the adaptor protein TRIF through the TIR domain of TLR4, thereby activating TBK1 and IKKε. These kinases subsequently phosphorylate IRF3 at Ser396, promoting its dimerization and nuclear translocation [30,89,90,91]. In the cGAS-STING pathway, DNA damage or oxidative stress-induced release of mtDNA into the cytosol within plaque cells is recognized by cGAS, which catalyzes cGAMP synthesis. cGAMP binds to STING and activates the TBK1-IRF3 axis [92,93]. Furthermore, a 2024 study revealed that STING in endothelial cells can also activate the PERK-eIF2α-ATF4 pathway via a non-canonical STING-PERK axis, forming a complex with IRF3 and NF-κB to synergistically promote pro-inflammatory gene expression [94].

Upon activation, IRF3 compromises endothelial barrier function by regulating Bax upregulation and Bcl-2 downregulation, thereby inducing endothelial cell apoptosis [95]; by promoting VE-cadherin internalization, increasing endothelial permeability [96]; and by activating NF-κB signaling to upregulate tissue factor (TF) and downregulate thrombomodulin (TM), disrupting the coagulation-anticoagulation balance and increasing thrombotic risk [97,98]. In ApoE^−/−^ mice, endothelial cell-specific IRF3 knockout significantly reduces atherosclerotic lesion area (−45%), decreases intraplaque macrophage infiltration, increases fibrous cap thickness, and enhances collagen deposition [99].

Additionally, IRF3 activation induces IFN-β production, which activates the JAK1-Tyk2-STAT1/2 pathway via autocrine and paracrine signaling. This pathway subsequently forms the ISGF3 complex with IRF9, further upregulating the expression of IRF3 and ISGF3 components (STAT1, STAT2, IRF9), thereby establishing a positive feedback loop termed the “IRF3-IFN-β-JAK-STAT-IRF3” circuit [100,101]. This feedback mechanism allows endothelial inflammation to self-perpetuate once initiated, providing a molecular explanation for the chronic nature of vascular inflammation in atherosclerosis.

IRF3-mediated endothelial dysfunction is a critical trigger for cardiovascular events. IRF3 activation promotes a pro-thrombotic state and thrombus formation by inducing endothelial cell apoptosis (via Bax upregulation) and inflammatory responses (via increased ICAM-1 expression) [99]. Consequently, targeting IRF3 may represent a potential strategy for preventing acute coronary thrombotic events through the preservation of vascular barrier function.

### 3.4. IRF5/IRF4

Phenotypic switching of macrophages within atherosclerotic plaques is a key determinant of lesion progression and regression. IRF5 and IRF4 constitute a functionally antagonistic regulatory system: IRF5 drives pro-inflammatory M1 polarization, whereas IRF4 promotes anti-inflammatory M2 polarization. The dynamic balance between these two factors contributes to determining plaque fate [30,102,103].

IRF5 is a key driver of macrophage pro-inflammatory polarization. Upon recognition of pathogen-associated molecular patterns (PAMPs) or damage-associated molecular patterns (DAMPs) by TLR4, the adaptor protein TRAF6 is recruited. TRAF6 undergoes K63-linked ubiquitination and activates the IKK complex (IKKα/IKKβ/NEMO), which in turn phosphorylates the C-terminal regulatory domain of IRF5, promoting its nuclear translocation and binding to target gene promoters [104,105]. Within the nucleus, IRF5 directly binds to the promoters of pro-inflammatory genes such as TNF-α, IL-1β, IL-6, and CCL2, driving M1 macrophage polarization. IRF5 also suppresses M2 conversion through a dual mechanism: it directly inhibits the transcriptional activity of PPARγ and STAT6, blocking the expression of M2 markers; and it recruits additional monocytes via the CCL2-CCR2 axis, establishing a positive feedback loop of “IRF5 → inflammation amplification → monocyte infiltration → further IRF5 activation” that perpetuates the inflammatory response [106]. At the level of lipid metabolism, IRF5 promotes cholesterol homeostasis imbalance by modulating the ABCA1/CD36 ratio: IRF5 reduces ABCA1 expression, decreasing cholesterol efflux, while upregulating the scavenger receptor CD36, increasing oxLDL uptake. This dual action results in substantial intracellular lipid accumulation and promotes foam cell formation [107,108].

In contrast to the pro-inflammatory role of IRF5, IRF4 functions as an antagonist of IRF5, primarily exerting anti-inflammatory and pro-resolving effects in macrophages. IRF4 synergizes with PPARγ to promote the expression of M2 markers Arg1, Ym1, and Fizz1 (i.e., Retnla) [106,109,110]. IRF4 also suppresses pro-inflammatory gene transcription by inhibiting NF-κB signaling, thereby promoting macrophage polarization toward the M2 phenotype from both directions, facilitating inflammation resolution and tissue repair. Regarding cholesterol metabolism, IRF4 may influence the LXRα signaling pathway, promoting the expression of cholesterol reverse transporters ABCA1 and ABCG1, enhancing cholesterol efflux from foam cells and reducing lipid accumulation [102,109,111]. Furthermore, IRF4 may participate in regulating the expression of efferocytosis receptors (e.g., MertK, Axl, Tim-4), enhancing the ability of macrophages to clear apoptotic cells. Timely clearance of apoptotic cells within plaques limits secondary necrosis and the spread of inflammation, thereby contributing to reducing necrotic core size and stabilizing plaque structure [112]. However, direct regulation of efferocytosis receptors by IRF4 requires further validation using gene knockout/overexpression experiments.

Based on the functional antagonism between IRF5 and IRF4, the IRF5/IRF4 ratio theoretically reflects the pro-inflammatory/anti-inflammatory balance of macrophages. Single-cell sequencing has identified a macrophage subpopulation with high IRF5 expression within atherosclerotic plaques [113], whereas IRF4 is a core driver of M2 polarization [109], suggesting that an elevated IRF5/IRF4 ratio may indicate M1 dominance and impaired inflammation resolution. PPARγ agonists (e.g., rosiglitazone) alleviate atherosclerotic lesions by synergizing with IRF4 to promote M2 polarization [111]. Theoretically, changes in this ratio could reflect treatment responses; however, its direct association with plaque stability, IMT improvement, or MACE risk requires clinical validation.

Based on the above mechanisms, it is plausible that an elevated IRF5/IRF4 ratio may reflect intraplaque M1/M2 imbalance and be associated with the risk of acute coronary syndrome (ACS). However, no clinical studies have directly measured the IRF5/IRF4 ratio in the peripheral blood of ACS patients. Its correlation with hs-CRP or cTnI, as well as its predictive value, awaits validation in prospective cohort studies. Restoring the balance of the IRF5/IRF4 ratio may represent a potential strategy for stabilizing plaques and preventing cardiac events.

### 3.5. IRF7/IRF8

IRF7 and IRF8 serve as terminal regulators in the late stage of vascular inflammation, driving plaque instability from the perspectives of vascular smooth muscle cell (VSMC) plasticity and adaptive immunity, respectively. Together, they constitute the molecular basis for the rupture of advanced atherosclerotic plaques.

IRF7 functions as a master switch governing the transdifferentiation of VSMCs towards a pro-inflammatory macrophage-like phenotype. A 2025 single-cell sequencing study revealed the existence of an IRF7^+^ VSMC subpopulation within atherosclerotic plaques. These cells co-express the SMC lineage marker CD200 and the macrophage marker CD68, exhibiting a pro-inflammatory phenotype. Lineage tracing experiments confirmed that ApoE^−/−^ mice with SMC-specific Irf7 knockout displayed reduced plaque area, a smaller lipid core, a thicker fibrous cap, and significantly enhanced plaque stability. Clinicopathological studies further support the clinical relevance of IRF7: IRF7 expression is significantly upregulated in unstable and advanced human atherosclerotic plaques, showing a strong positive correlation with inflammatory macrophage burden. Moreover, CD200^+^/IRF7 high SMC-derived macrophage-like cells are significantly enriched in symptomatic plaques, suggesting that IRF7-driven VSMC transdifferentiation is closely linked to clinical events [114].

In contrast to the predominant role of IRF7 in innate inflammatory signaling, IRF8 contributes to adaptive immune regulation, particularly through its involvement in dendritic cell (DC) development and function. IRF8 is a critical transcription factor for the development of CD11b^−^CD103^+^ conventional DC type 1 (cDC1s). These cDC1s possess potent antigen-presenting capabilities, effectively activating naive T cells and initiating and amplifying T cell-mediated adaptive immune responses [25]. In an atherosclerosis model, conditional knockout of IRF8 in CD11c^+^ cells led to the loss of CD11b^−^CD103^+^ cDC1s and CD8α^+^ cDCs, significantly reduced T/B cell activation and differentiation, and substantially attenuated atherosclerotic lesions. This confirms the pivotal role of IRF8-dependent DC subsets in adaptive immunity [115]. Furthermore, as a downstream target gene of the IFN-γ/JAK2/STAT1 pathway, IRF8 responds to inflammatory signals to regulate macrophage function and promote the release of inflammatory cytokines [116]. IRF8 is also involved in regulating the resolution of macrophage inflammation: IRF8-deficient macrophages exhibit reduced CD36 expression, impairing their efferocytosis of apoptotic neutrophils, which leads to persistent inflammation and plaque instability [117].

IRF7 and IRF8 are also intricately linked to the “aging” characteristics of vascular inflammation. Senescent endothelial cells and VSMCs secrete senescence-associated secretory phenotype (SASP) factors, including IL-6 and IL-8. These factors activate the cGAS-STING pathway in neighboring cells via paracrine signaling. Concurrently, telomere shortening in senescent cells induces a DNA damage response (DDR). The DDR, in turn, activates the cGAS-STING-IRF3 axis, promoting type I interferon production and the release of more SASP factors, thereby forming a vicious “inflammation-aging” cycle [25]. Furthermore, IRF8 exerts an independent role in cellular senescence: in a DNA damage-induced senescence model, IRF8 deletion significantly suppresses the expression of SASP genes, whereas overexpression of wild-type IRF8 restores the senescent phenotype [90]. These findings suggest that IRF8 may contribute to the persistence of inflammatory signaling during advanced vascular disease, although the clinical implications of this pathway for cardiovascular events remain to be elucidated.

IRF7 and IRF8 jointly contribute to the progression of advanced plaques toward acute cardiac events. IRF7^+^ vascular smooth muscle cells (VSMCs) may compromise fibrous-cap integrity through maladaptive phenotypic remodeling, whereas IRF8^+^ conventional dendritic cell type 1 (cDC1s) sustain local adaptive immune activation. Clinical autopsy studies have shown that the density of CD200^+^/IRF7high cells in ruptured plaques from sudden death patients is significantly higher than in stable plaques and is positively correlated with the thrombus burden score. Additionally, IRF8 expression levels rise in parallel with intraplaque CD4^+^ T cell infiltration density and serum hs-CRP levels. The combined effect of IRF7-mediated structural destruction and IRF8-mediated inflammation maintenance renders advanced plaques prone to rupture under stress, thereby contributing to myocardial infarction and other acute cardiovascular events. Therefore, a combined intervention targeting IRF7/8, such as SMC-specific IRF7 silencing combined with cDC1-targeted IRF8 blockade, could simultaneously enhance fibrous cap stability and suppress the inflammatory response, offering a new strategy for preventing myocardial infarction [114,118] (Figure 1 and Table 1).

### 3.6. Integrative Regulation and Precision Intervention of the IRF Family in Vascular Inflammation

Within the vascular inflammatory microenvironment, IRFs do not function in isolation but constitute a complex regulatory network with systems biology characteristics. In this network, IRF1, IRF3, IRF5, and IRF7 form a tightly connected pro-inflammatory core, mutually amplifying each other through positive feedback loops to continuously drive the progression of vascular inflammation. In contrast, IRF4 acts as an anti-inflammatory node, connecting pathways such as PPARγ and STAT6 to limit excessive inflammatory activation. Network analysis further reveals that IRF3 and IRF5 occupy hub positions in the regulatory network, possessing the highest degree of connectivity and intervention efficiency, making them ideal entry points for precise targeting. However, this network also exhibits significant robustness-knockout of a single IRF is often compensated for by other family members. For instance, IRF7 can be compensatorily upregulated following IRF3 deletion. This characteristic explains the limitations of targeting a single IRF in clinical applications and provides a theoretical basis for designing combined intervention strategies [102,119,120].

A stage-specific precision targeting strategy demonstrates significant clinical value. In the early stage of vascular inflammation, endothelial injury and monocyte recruitment are primarily driven by IRF3. Employing an IRF3 inhibitor (e.g., Amlexanox) at this stage can effectively protect the endothelial barrier and block the initiation of inflammation [121,122]. In the middle stage, macrophage infiltration and M1 polarization become the dominant pathological processes. IRF5 promotes the release of pro-inflammatory cytokines and foam cell formation. Applying PPARγ agonists at this stage can modulate the IRF5/IRF4 ratio, inhibiting macrophage pro-inflammatory polarization and promoting cholesterol efflux [123]. In the late stage, VSMC transdifferentiation and plaque instability become the primary issues. IRF8 sustains adaptive immune responses through DC subsets. Regulating IRF8 activity with JAK2 inhibitors at this stage can help stabilize the fibrous cap [124].

The clinical translation of IRF-targeted therapies is heavily reliant on support from precise delivery systems. Endothelial-targeted nanoparticles, modified with VCAM-1 antibodies, can specifically deliver drugs to activated endothelial cells [58], achieving plaque endothelial enrichment. Macrophage-targeted liposomes, modified with markers like SR-A, can precisely deliver drugs to plaque macrophages, enabling specific regulation of inflammatory cells [125]. Engineered exosomes loaded with miR-146a mimics utilize the natural targeting ability of exosomes to inhibit TLR signaling-mediated inflammatory responses via the IRAK1/TRAF6 pathway [126]. Furthermore, pH-sensitive or ROS-sensitive nanocarriers can specifically release drugs within the acidic or high oxidative stress microenvironment of plaques. pH-responsive hyaluronic acid nanoparticles target plaques through HA-CD44 interaction and release drugs in the acidic inflammatory microenvironment. ROS-responsive nano-prodrugs, linked by thioketal bonds, release active statins in the high-ROS environment of plaques, synergistically enhancing the anti-atherosclerotic effect [74] (Table 2). Whether eicosanoid signaling constitutes an additional upstream metabolic-inflammatory input or a downstream effector interacting with IRF-dependent transcriptional programs remains to be determined.

## 4. The Regulatory Network of IRFs in Pan-Vascular Inflammatory Diseases

Atherosclerosis, as a local manifestation of systemic vascular inflammation, shares significant pathological commonalities in its molecular regulatory mechanisms with diseases such as hypertension, ACS, heart failure, and aortic aneurysm/dissection. Within this disease spectrum, the IRF family does not function in isolation. Instead, it constitutes a molecular hub for trans-organ vascular inflammation through conserved innate immune signaling axes (TLR/cGAS-STING-IRF3/7) and adaptive immune regulatory nodes (IRF4/8). This section systematically elaborates on the regulatory principles and clinical translational value of IRFs in pan-cardiovascular diseases, using IRF functional modules as the framework and the disease spectrum as the context.

### 4.1. Hemodynamic Stress and IRF-Driven Vascular Remodeling: A Model of Hypertension

The essence of hypertensive vasculopathy is chronic inflammation and structural remodeling of the vessel wall triggered by hemodynamic stress. In this pathological context, the same IRF member can exhibit completely opposite biological effects depending on the cell type, microenvironment, and signaling pathway. IRF1 serves as a typical example of such context-dependent regulation.

In VSMCs, IRF1 counteracts the excessive proliferative signals mediated by the angiotensin II type 1 receptor (AT1R) by upregulating the angiotensin II type 2 receptor (AT2R). Under Ang II stimulation, IRF1 synergizes with Klf-5 to activate AT2R transcription, inducing VSMC apoptosis and inhibiting neointimal formation. In a spontaneously hypertensive rat (SHRSP) model, decreased IRF1 expression parallels reduced AT2R expression, and transfection with IRF1 antisense oligonucleotides further suppresses AT2R expression [75,78,79]. This mechanism suggests that in the early stages of hypertension, the IRF1-AT2R axis constitutes an endogenous negative feedback loop counteracting excessive Ang II/AT1R activation, exerting a vascular protective effect.

However, the protective role of IRF1 is strictly dependent on cell type and disease stage. When pathological stimuli exceed the compensatory threshold (e.g., persistent oxidative stress or ox-LDL exposure), IRF1 shifts to a pro-inflammatory phenotype in macrophages and endothelial cells. It drives VSMC towards a pathological phenotype by activating CCL19 transcription [78] or mediates endothelial cell pyroptosis via the GSDMD/caspase-1 pathway [79,80]. This duality is not a functional contradiction of IRF1 but an inherent characteristic of its role as a stress-responsive transcription factor-its transcriptional output depends on the co-activator profile, DNA accessibility, and upstream signal intensity. Therefore, in patients with hypertension complicated by atherosclerosis or metabolic syndrome, the net effect of IRF1 may shift from protective to pro-inflammatory, a transition point worthy of clinical attention.

Unlike the context-dependent role of IRF1, IRF3 primarily exerts a pro-inflammatory amplifying effect in hypertensive vascular disease. Persistent hemodynamic stress-induced mitochondrial damage can release mtDNA, activating the cGAS-STING-TBK1-IRF3 signaling axis and driving the sustained transcription of type I interferons and chemokines (e.g., RANTES) [89,92]. Notably, IRF3 overactivation possesses self-perpetuating properties: its downstream product, IFN-β, can further upregulate IRF3 expression via the JAK-STAT positive feedback loop [100,101], forming a vicious “inflammation-remodeling” cycle. This mechanism is conserved across various hypertensive complications (including target organ damage and vascular fibrosis), suggesting that the IRF3 pathway may be a molecular switch connecting hemodynamic stress and vascular inflammation.

Research on IRF7 and IRF8 in hypertension is still in the exploratory phase. As the master regulator of TLR7/9-mediated type I interferon responses, IRF7 may participate in hemodynamic stress-induced immune activation within the vessel wall [114]. IRF8 drives macrophage polarization towards an M2-like phenotype via S1P1 receptor signaling, while being precisely regulated by epigenetic modifications (e.g., H3K27me3) [25,116]. Although direct evidence is limited, based on IRF8’s central role in macrophage plasticity, it is hypothesized that it may be involved in the dynamic balance between inflammation resolution and fibrotic repair during hypertensive vascular remodeling.

### 4.2. IRF Regulation in Plaque Instability and Thrombosis: The Molecular Basis of Acute Coronary Syndrome

The pathological core of ACS lies in an uncontrolled activation of local inflammatory responses and the coagulation cascade following atherosclerotic plaque rupture or erosion. The roles of IRFs in this process exhibit significant spatiotemporal specificity: IRF1/IRF5 dominate the cellular basis of plaque destabilization, while IRF3/IRF7 connect tissue damage to systemic inflammatory responses.

IRF1 is a key intracellular marker of plaque vulnerability. Human autopsy studies have demonstrated that IRF1 expression in the fibrous cap of unstable plaques is significantly higher than in stable plaques, and is positively correlated with lipid core area and macrophage apoptosis index [86,87]. Mechanistically, IRF1 attenuates plaque stability through dual pathways: in macrophages, it drives the transcription of pro-inflammatory mediators (TNF-α, IL-1β, MMPs) and accelerates apoptosis; in VSMCs, it promotes their transition to a pro-inflammatory, synthetic phenotype, reducing collagen secretion [78,86,87]. Notably, the plaque-stabilizing effect of statins is partly attributable to their transcriptional inhibition of IRF1; atorvastatin can significantly reduce IRF1 expression and transcriptional activity in human monocytes/macrophages, offering a novel molecular explanation for “pleiotropic benefits beyond lipid-lowering” [88].

The imbalance of the IRF5/IRF4 axis constitutes the genetic and transcriptional basis of the ACS inflammatory phenotype. Carriers of the IRF5 risk allele (rs2004640 T) exhibit high IRF1 expression in monocytes, an elevated M1/M2 ratio, and increased levels of TNF-α/iNOS, with a significantly higher risk of coronary heart disease [104,105]. Plaque biopsies from ACS patients show that the density of IRF5^+^ M1 macrophages is positively correlated with thrombus burden scores [106]. Conversely, low IRF4 expression is independently associated with increased plaque burden and a higher risk of major adverse cardiac events (MACE) [110]. Single-cell comparative analyses further reveal that stable plaques exhibit an IRF4”high”/IRF5”low” pattern, accompanied by M2-like macrophage infiltration and a thick fibrous cap; unstable plaques, in contrast, display an IRF4”low”/IRF5”high” pattern, with M1 macrophage predominance and a thin fibrous cap [108]. A meta-analysis of genome-wide association studies (GWAS) has confirmed that variants in the IRF4 gene region are associated with protection from coronary heart disease, while variants in the IRF5 region are associated with risk, with the effects partly mediated by differential monocyte methylation [107]. This evidence establishes the IRF4/IRF5 axis as a core regulatory node linking genetic susceptibility, macrophage polarization, and plaque stability.

The cGAS-STING-IRF3 signaling axis constitutes a pathological bridge connecting plaque rupture, local inflammation, and thrombosis in ACS. Genomic DNA and mtDNA released upon plaque rupture act as DAMPs, recognized by the cytosolic DNA sensor cGAS, which catalyzes cGAMP synthesis, thereby activating the STING-TBK1-IRF3 pathway [92,93]. The pathological significance of this pathway is multifaceted: IRF3 activation not only upregulates adhesion molecules like ICAM-1 to promote platelet adhesion and leukocyte recruitment [95], but also disrupts the coagulation-anticoagulation balance by inducing tissue factor (TF) expression and downregulating thrombomodulin (TM) [97,98]. Furthermore, mtDNA-cGAS-STING signaling can promote macrophage pyroptosis through synergistic IRF3/IRF7 activation, forming a positive feedback loop of “injury-inflammation-thrombosis” [114]. In terms of targeted intervention, compounds such as cinnamic acid can directly bind to STING and block its interaction with IRF3, inhibiting downstream signaling [96]; similarly, total glucosides of paeony can alleviate cGAS-STING-mediated vascular inflammation through analogous mechanisms [97]. These findings provide a theoretical basis for synergistically inhibiting vascular inflammation alongside antiplatelet/anticoagulant therapy.

The role of IRF7 in ACS has dual interpretative dimensions. On one hand, macrophages within plaques specifically inhibit IRF7-dependent TLR9 responses via the BAFF-TACI signaling pathway, negatively regulating the production of pro-atherogenic factors such as CXCL10 [114]. This suggests that dysregulation of the IRF7 pathway may be an intrinsic driver of exacerbated plaque inflammation. On the other hand, dynamic monitoring of serum IRF7 or its downstream effectors (e.g., CXCL10) may serve as circulating biomarkers for identifying high-risk plaques [114]. However, the predictive value of IRF7 as a biomarker necessitates validation in prospective cohort studies.

### 4.3. IRF Regulation in Myocardial Remodeling and Fibrosis: From Ischemic Injury to Heart Failure

The pathological progression of heart failure (HF) involves a cascade of cardiomyocyte death, fibroblast activation, and extracellular matrix deposition. IRFs primarily act through two pathways in this spectrum: IRF3-mediated innate immune signaling drives myocardial fibrosis, while IRF1/IRF7 participate in the amplification of inflammation following ischemic injury.

IRF3 is a core transcriptional regulator of myocardial fibrosis. Following myocardial infarction (MI), double-stranded DNA (dsDNA) released from necrotic cardiomyocytes activates the cGAS-STING-IRF3 pathway in infiltrating macrophages, inducing a type I interferon response and promoting the conversion of fibroblasts into myofibroblasts; the selective STING inhibitor H-151 can significantly improve cardiac function and reduce fibrosis by blocking this pathway [99]. In a dilated cardiomyopathy model induced by LMNA gene mutations, persistent DNA damage response (DDR) in cardiomyocytes activates cGAS. Genetic ablation of cGAS (via Mb21d1 knockout) prolongs survival, improves cardiac function, and reduces fibrosis [101]. Although this study did not directly measure IRF3 activity, given that IRF3 is the canonical downstream effector of the cGAS-STING pathway, this finding indirectly supports the pathogenic role of IRF3 in hereditary cardiomyopathy. Furthermore, analysis of pharmacovigilance databases shows that in patients experiencing heart failure associated with VEGF/VEGFR inhibitors, the upregulation of IRF3/IRF7-mediated immune response pathways occurs concurrently with increases in classical inflammatory cytokines (IL-1, IL-6, TNF-α) [98], suggesting that IRF3 pathway activation may be a common molecular basis for chemotherapy-related cardiotoxicity.

IRF1 plays dual pro-apoptotic and pro-inflammatory roles in myocardial ischemia/reperfusion (MIR) injury. IRF1 expression is significantly upregulated in the hearts of mice subjected to ischemia/reperfusion. Cardiac-specific knockout of IRF1 reduces infarct size, improves cardiac function, and suppresses cardiomyocyte apoptosis [86]. Mechanistically, IRF1 exacerbates oxidative stress through transcriptional regulation of inducible nitric oxide synthase (iNOS), forming an “IRF1-iNOS-ROS-cell death” cascade [87]. This mechanism is molecularly consistent with the pro-inflammatory effects of IRF1 in atherosclerotic plaques, suggesting that IRF1 may represent a common therapeutic target for ischemic cardiovascular diseases.

Research on IRF7 in heart failure has revealed its systemic regulatory functions that extend beyond local inflammation. Single-nucleus RNA sequencing analysis has identified IRF7 as a key mediator of the cardioprotective immunomodulatory effects of drugs like sacubitril/valsartan in diabetic cardiomyopathy [25]. In vitro experiments confirm that directly inhibiting IRF7 expression can suppress the activation of cardiac fibroblasts by pro-inflammatory macrophages, thereby inhibiting a key step in myocardial fibrosis [115]. In clinical cohorts, peripheral blood NT-proBNP and CRP levels rise synchronously in patients treated with VEGF(R) inhibitors; although IRF7 was not directly measured, the tight association of its pathway activation with systemic inflammation and myocardial injury provides logical support for IRF7 as a circulating biomarker [114]. In summary, IRF7 is not merely a product of local inflammation but also acts as a systemic signaling molecule involved in the entire process from metabolic dysregulation to myocardial remodeling, and its targeted intervention holds potential for broad-spectrum cardiovascular protection. Recent reviews have further summarized the broader role of IRFs in ischemia/reperfusion injury across multiple organs, highlighting dynamic regulatory networks and organ-specific IRF axes [31]. The present review focuses specifically on their implications for cardiovascular remodeling and inflammation.

### 4.4. IRF Mechanisms in Aortic Wall Homeostasis Disruption: Aortic Aneurysm and Dissection

The pathological hallmark of aortic aneurysm and dissection (AAD) is the destruction of the aortic medial layer structure, degradation of the extracellular matrix (ECM), and intramural inflammatory infiltration. The role of IRFs in this process centers on VSMC phenotypic transformation and immune microenvironment remodeling, with epitranscriptomic regulation by IRF3 being particularly prominent.

IRF3 drives pathological VSMC phenotypic transformation through epigenetic reprogramming. Single-cell RNA sequencing has revealed that aortic stress induces a transition of VSMCs from a contractile phenotype to proliferative, ECM-producing, and inflammatory phenotypes. Lineage tracing confirms that VSMCs can fully convert into fibroblast- and macrophage-like cells [119]. Mechanistically, after cytosolic DNA leakage in the aortic wall activates the STING-TBK1-IRF3 axis, activated IRF3 binds and recruits EZH2 to the promoter regions of contractile genes (e.g., MYH11, ACTA2). This induces the repressive histone modification H3K27me3, leading to silencing of the contractile phenotype gene program and a shift towards a pro-inflammatory, pro-proliferative pathological phenotype [120]. Genetic evidence indicates that global or conditional knockout of Sting or Irf3 can inhibit VSMC inflammatory phenotype transformation, protect the SMC population, and reduce macrophage pro-inflammatory programming and aortic wall destruction. This effectively delays aneurysm progression and lowers the incidence of dissection [121]. This mechanism positions IRF3 as a molecular hub linking DNA damage sensing, epigenetic remodeling, and cell fate determination. Its therapeutic relevance is not limited to AAD but is likely conserved in multiple cardiovascular diseases, including hereditary cardiomyopathy, hypertension, and atherosclerosis [122].

Regulation of IRF1 in aortic dissection involves a non-coding RNA network. In an Ang II-induced aortic dissection model, IRF1 is negatively regulated as a downstream target gene of miR-650. miR-650 targets IRF1, thereby negatively regulating TGFβR1 expression, promoting the proliferation and migration of human aortic VSMCs, and inhibiting the expression of contractile phenotype markers [79]. Conversely, circ_0022920 can sponge miR-650, upregulating IRF1 and TGFβR1 expression, thus maintaining the VSMC contractile phenotype and inhibiting dissection progression [79]. This mechanism suggests that oxidative stress-induced overactivation of IRF1 might be a precipitating factor in aortic wall injury. Theoretically, antioxidant strategies capable of effectively modulating IRF1 signaling could attenuate vascular injury. However, this deduction currently lacks direct in vivo interventional evidence and requires further validation.

### 4.5. Cross-Disease Integration: IRFs as Common Regulatory Nodes in Vascular Inflammation

Synthesizing the findings from the four cardiovascular disease models above, the regulatory patterns of IRFs exhibit significant modularity and context-dependency.

The pro-inflammatory core module (IRF1/IRF3/IRF5/IRF7) is activated across multiple diseases through the conserved cGAS-STING-TBK1 axis or the TLR-TRAF6-IKK axis, driving type I interferon responses, transcription of pro-inflammatory cytokines, and immune cell recruitment. The common pathological consequence is the disruption of the “injury-repair” dynamic balance, shifting the inflammatory response from adaptive protection to chronic self-perpetuation. Notably, the target cell spectrum of the same IRF member varies among diseases-IRF1 primarily acts on macrophages and VSMCs in AS but preferentially regulates AT2R expression in VSMCs in hypertension; IRF3 disrupts the endothelial barrier in AS but reprograms VSMC phenotype in AAD. This cell-type preference dictates that targeted interventions require consideration of disease-specific delivery strategies.

The anti-inflammatory/regulatory module (IRF4/IRF8) displays relatively conserved functions, primarily promoting inflammation resolution by driving M2-like macrophage polarization, enhancing reverse cholesterol transport (IRF4), or regulating dendritic cell subset development and efferocytosis (IRF8). The IRF4/IRF5 ratio, as an integrative indicator of macrophage pro-inflammatory/anti-inflammatory balance, correlates with plaque stability and clinical prognosis in both AS and ACS [103,108,110], suggesting its potential as a cross-disease biomarker of inflammatory burden.

Clinical Translational Implications: The cross-disease conservation of IRFs provides a rationale for developing broad-spectrum cardiovascular anti-inflammatory drugs. However, it also necessitates precise discrimination based on disease stage and target cell type. For instance, while systemic IRF3 inhibition could mitigate myocardial fibrosis in AAD and HF, it might compromise antiviral immunity; IRF5 antagonism promotes plaque stabilization in AS but could impair pathogen clearance in infectious diseases. Therefore, lesion-specific intervention employing targeted delivery systems (e.g., VCAM-1 antibody-modified nanoparticles, SR-A-targeted liposomes, or ROS/pH-sensitive carriers) [74,125,127] represents a critical pathway for the clinical translation of IRF-targeted therapies (Table 3).

## 5. Summary and Perspectives

### 5.1. IRFs: The “Molecular Compass” of Vascular Inflammation

This review systematically elucidates the central role of the IRF family in vascular inflammation, using AS, the most representative cardiovascular disease, as the primary model. It reveals the multidimensional regulatory function of IRFs as a “molecular compass.” IRFs are not simple pro-inflammatory or anti-inflammatory switches; rather, they are finely tuned regulators that dynamically shift their function based on cell type, microenvironmental signals, and disease stage. This dual nature stems from their transcriptional plasticity conferred by a conserved DNA-binding domain and a variable C-terminal regulatory domain [21,22], as well as their pivotal position at the intersection of multiple signaling pathways [23]. The dynamic balance between the pro-inflammatory core constituted by IRF1/IRF3/IRF5/IRF7 and the anti-inflammatory network mediated by IRF4/IRF8 determines the trajectory of vascular inflammation from adaptive protection to chronic self-sustenance. This principle is well-validated in cardiovascular diseases, exemplified by the progression of AS plaques from a stable to a vulnerable state [26,27,28,29]. Critically, IRFs establish an “epigenetic memory” of vascular inflammation by integrating innate immune signaling (TLR/cGAS-STING), metabolic reprogramming (glycolysis/fatty acid oxidation), and epigenetic modifications (DNA methylation/histone acetylation), enabling local inflammatory responses to become self-perpetuating and resistant to conventional anti-inflammatory therapies [66,67,68,69,70,71]. This characteristic profoundly explains why traditional broad-spectrum anti-inflammatory strategies have yielded limited success in preventing and treating cardiovascular diseases and provides the theoretical rationale for targeted intervention against IRFs. Accordingly, the principal contribution of this review is not to re-summarize the canonical functions of individual IRFs, but to integrate their cell- and stage-dependent activities into a cross-disease framework of vascular inflammation and to translate this framework into potential therapeutic windows and delivery strategies.

### 5.2. From Mechanism to Translation: Clinical Pathways for Precision Intervention

Despite this promise, the clinical translation of IRF-targeted therapies for cardiovascular diseases faces multiple formidable challenges. First, the IRF regulatory network exhibits significant robustness; genetic deletion of a single IRF member is often buffered by functional compensation from other family members. For instance, knockout of IRF3 can lead to compensatory upregulation of IRF7 to maintain type I interferon responses [119,120]. This redundancy suggests that interventions targeting a single IRF may be insufficient to break the vicious cycle of cardiovascular inflammation. Future directions may necessitate combination targeting strategies or pan-IRF modulators (e.g., simultaneously suppressing the IRF1/3/5/7 pro-inflammatory axis while enhancing IRF4/8 anti-inflammatory functions). Second, the physiological roles of IRFs in antiviral immunity and tumor surveillance cannot be ignored; systemic IRF inhibition could increase the risk of infection and compromise immune surveillance [24]. Therefore, there is an urgent need to develop cardiovascular lesion-specific delivery systems, such as VCAM-1 antibody-modified nanoparticles, SR-A-targeted liposomes, or ROS/pH-sensitive carriers, to achieve drug enrichment and controlled release at vascular inflammatory foci [74,125,127]. Finally, most current IRF research remains confined to AS animal models and in vitro experiments, lacking large-scale clinical cohort validation across different diseases. Future prospective studies are required to establish quantitative relationships between IRF expression levels, imaging biomarkers of vascular inflammation (e.g., carotid intima-media thickness, vascular wall ^18^F-FDG uptake), and the risk of major adverse cardiovascular events (MACE). This will enable the establishment of an IRF profiling-based personalized risk stratification system for cardiovascular diseases.

### 5.3. Cross-Disease Perspective: IRFs as a Universal Regulatory Node of Vascular Inflammation

Extending beyond AS, IRFs play critical roles across a spectrum of cardiovascular diseases, including hypertensive vascular remodeling, ACS, HF, and AAD, suggesting they may serve as a universal molecular node linking vascular inflammation to cardiac events. In hypertension, hemodynamic stress drives chronic inflammation and fibrosis of the vascular wall via the cGAS-STING-IRF3 axis, while the IRF1-AT2R axis plays a protective, negative-feedback role in the early stages [75,78,89]. In ACS, DAMPs released upon plaque rupture or erosion activate the cGAS-STING-IRF3 axis, forming a pathological bridge connecting localized vascular inflammation amplification to acute thrombotic cardiac events [92,93]. In heart failure, dsDNA released from necrotic cardiomyocytes activates the cGAS-STING-IRF3/IRF7 pathway, driving myocardial fibrosis and ventricular remodeling, which are structural cardiac pathologies [98,99]. In AAD, DNA damage within the aortic wall induces epigenetic reprogramming via the STING-TBK1-IRF3 axis, leading to the loss of the contractile phenotype in vascular smooth muscle cells and structural destruction of the vessel wall [120,121]. Despite differences in target cell spectra and tissue microenvironments, the antagonistic balance between the pro-inflammatory core (IRF1/IRF3/IRF5/IRF7) and the anti-inflammatory module (IRF4/IRF8) constitutes a common regulatory framework for the aforementioned cardiovascular diseases. This cross-disease conservation suggests that IRF-targeted interventions possess broad-spectrum potential for regulating cardiovascular pathologies. However, it also mandates careful consideration of disease-specific microenvironmental differences and cell-type preferences in therapeutic strategy design to avoid off-target effects from a one-size-fits-all approach.

### 5.4. Future Research Directions

Recent work on IRF signaling in ischemia/reperfusion injury has further proposed dynamic regulatory-network and organ–IRF axis frameworks, highlighting the potential of single-cell sequencing, organoid modeling, artificial intelligence-assisted prediction, and organ-specific delivery for resolving the context dependence of IRF function [31]. These approaches may be particularly valuable for cardiovascular applications, where the same IRF member may exert divergent effects across vascular, myocardial, and immune compartments. Looking ahead, IRF research in the context of cardiovascular diseases should strive for breakthroughs in the following dimensions: (1) Single-cell resolution IRF regulation atlas: Utilize single-cell RNA sequencing and spatial transcriptomics to map the expression heterogeneity of IRFs across different cell subpopulations within cardiovascular lesions and delineate intercellular regulatory networks. This will identify key cell–IRF pairs driving cardiovascular inflammation progression and establish a common atlas alongside disease-specific signatures for a range of cardiovascular diseases (AS, hypertension, HF, AAD). (2) Post-translational modifications and metabolic crosstalk of IRFs: Deeply investigate the roles of post-translational modifications (PTMs) of IRFs, such as phosphorylation, ubiquitination, and acetylation, in regulating the metabolic reprogramming of cardiovascular immune cells. This will uncover novel regulatory mechanisms forming a trinity of immunity, metabolism, and epigenetics. (3) Clinical cardiovascular translation of IRF-targeted drugs: Develop highly selective IRF small-molecule inhibitors (e.g., TBK1/IKKε inhibitors, IRF5-specific antagonists) and biologics (e.g., anti-IRF7 monoclonal antibodies). Optimize their pharmacokinetic profiles using cardiovascular lesion-specific delivery systems to mitigate the risk of systemic immunosuppression. (4) Combination of IRF modulation with emerging therapeutic strategies: Explore synergistic applications of IRF regulation with gene editing (CRISPR-Cas9), RNA interference (siRNA/miRNA), and cell-based therapies (engineered exosomes) in the cardiovascular field. This will pave the way for constructing a multi-layered precision intervention system for cardiovascular diseases [126].

In conclusion, the IRF family, with its unique dual regulatory characteristics, extensive signal integration capability, and remarkable spatiotemporal expression heterogeneity, represents an ideal target for precision intervention in cardiovascular diseases. Using AS as a primary disease model, IRF research is driving a paradigm shift in the treatment of cardiovascular diseases from traditional “broad-spectrum anti-inflammation” towards “targeted rebalancing” based on molecular classification. While challenges remain, the deep integration of multi-omics technologies and the continuous innovation of cardiovascular lesion-specific delivery systems hold the potential for IRF-centered precision strategies of vascular inflammation to open new avenues for reducing the global burden of cardiovascular diseases.

## Figures and Tables

**Table 1:** Investigating the effect of IRF family in vascular inflammation.

IRF Member	Phenotype	References
IRF1	Pro-inflammatory hub: activates iNOS–NO/p21 and CCL19 in VSMCs; mediates GSDMD/Caspase-1 pyroptosis and VCAM-1 upregulation in ECs	[75,76,77,78,79,80,81]
	Drives M1 polarization via STAT1; promotes NLRP3 inflammasome assembly and PANoptosis in macrophages	[82,83,84,85,86,87]
IRF3	Central regulator of endothelial activation: TLR4-TRIF/cGAS-STING → TBK1/IKKε phosphorylation and nuclear translocation	[89,90,91,92,93]
	Establishes “IRF3-IFN-β-JAK-STAT” positive feedback loop; co-activates NF-κB to amplify inflammation	[94,95,96,97,98,99,100,101]
IRF5	Key driver of M1 polarization: TLR4-TRAF6-IKK → nuclear translocation; inhibits PPARγ/STAT6	[102,103,104,105,106]
	Shifts ABCA1/CD36 ratio: ↓ cholesterol efflux, ↑ oxLDL uptake, promotes foam cell formation	[107,108]
IRF4	Anti-inflammatory counterbalance: synergizes with PPARγ to drive M2 polarization (Arg1, Ym1, Fizz1)	[102,106,109]
	Enhances ABCA1/ABCG1-mediated cholesterol efflux; promotes efferocytosis (MertK, Axl, Tim-4)	[109,110,111]
IRF7	Master switch for VSMC transdifferentiation into macrophage-like phenotype (CD200^+^/CD68^+^)	[73,74]
IRF8	Orchestrates cDC1 development (CD11b^−^CD103^+^) for adaptive immunity; regulates macrophage efferocytosis	[25,117]
	Mediates cellular senescence via SASP	[115,116]

Note: VSMCs, vascular smooth muscle cells; ECs, endothelial cells; cDC1, conventional type 1 dendritic cells; SASP, senescence-associated secretory phenotype.

**Table 2:** Stage-specific IRF expression profiles and precision intervention strategies in AS.

Stage	Pathological Characteristics	Dominant IRF Mechanism	References
Early (fatty streak)	Endothelial activation, monocyte recruitment	Endothelial IRF3 induces adhesion molecules via TLR4-TRIF and cGAS-STING pathways	[66,72]
Middle (fibrous plaque)	Macrophage M1 polarization, foam cell formation	Macrophage IRF1/IRF5 drive M1 polarization; IRF4 anti-inflammatory effect suppressed	[28,69]
Late (complex lesion)	VSMC transdifferentiation, fibrous cap thinning	VSMC IRF7 drives macrophage-like transdifferentiation; DC IRF8 sustains adaptive immunity	[73,74]

Note: VSMC, vascular smooth muscle cell.

**Table 3:** Investigating the effect of IRF modules across cardiovascular diseases.

Disease Model	Dominant IRF Mechanism	References
Atherosclerosis	Stage-specific IRF3 → IRF1/5 → IRF7/8 progression	[72,74]
Hypertension	IRF1 context-dependent switching; IRF3 chronic amplification	[75,89]
Acute coronary syndrome	IRF1/5 plaque destabilization; IRF3/7 thrombosis bridge	[105,114]
Heart failure	IRF3/7 fibrosis; IRF1 ischemic injury	[87,99]

## Data Availability

No new data were created or analyzed in this study. Data sharing is not applicable to this review article.

## Abbreviations

The following abbreviations are used in this manuscript:

CVD	Cardiovascular disease
LDL-C	low-density lipoprotein cholesterol
ROS	reactive oxygen species
IRFs	Interferon Regulatory Factors
IRF	Interferon Regulatory Factor
DBD	DNA-binding domain
HTH	helix-turn-helix
ISRE	interferon-stimulated response element
IFN	interferon
TM	downregulate thrombomodulin
PAMPs	pathogen-associated molecular patterns
DAMPs	damage-associated molecular patterns
ACS	acute coronary syndrome
cDC1s	conventional DC type 1
DC	dendritic cel
SASP	senescence-associated secretory phenotype
DDR	DNA damage response
STEMI	ST-elevation myocardial infarction
AT1R	angiotensin II type 1 receptor
SHRSP	spontaneously hypertensive rat
GWAS	genome-wide association studies
TF	tissue factor
HF	heart failure
TM	thrombomodulin
MI	myocardial infarction
dsDNA	double-stranded DNA
MIR	myocardial ischemia/reperfusion
iNOS	inducible nitric oxide synthase
AAD	aneurysm and dissection
ECM	extracellular matrix
MACE	major adverse cardiovascular events
PTMs	post-translational modifications
